# Update on Prognostic and Predictive Markers in Mucinous Ovarian Cancer

**DOI:** 10.3390/cancers15041172

**Published:** 2023-02-12

**Authors:** Fulvio Borella, Marco Mitidieri, Stefano Cosma, Chiara Benedetto, Luca Bertero, Stefano Fucina, Isabelle Ray-Coquard, Annalisa Carapezzi, Domenico Ferraioli

**Affiliations:** 1 Gynecology and Obstetrics 1U, Departments of Surgical Sciences, City of Health and Science, University of Turin, 10126 Turin, Italy; 2 Gynecology and Obstetrics SC4, Departments of Surgical Sciences, City of Health and Science, 10126 Turin, Italy; 3 Pathology Unit, Department of Medical Sciences, University of Turin, 10126 Turin, Italy; 4 Leon Berard Comprehensive Cancer Center, 69008 Lyon, France; 5 Department of Surgical Sciences, University of Turin, 10126 Torino, Italy; 6 Hospital Group South Doors, 69200 Vénissieux, France

**Keywords:** mucinous ovarian cancer, ovarian cancer, prognosis, prognostic factors, target therapy, predictive factors, molecular features, surgery, survival, pathology

## Abstract

**Simple Summary:**

Mucinous ovarian cancer (MOC) is a rare tumor that can be classified according to stage in early MOC and advanced MOC. Early MOC is characterized by a good prognosis and, in most cases, surgical treatment alone represents the mainstay of therapy. Despite the good prognosis, some early-stage MOCs can relapse and exhibit aggressive behavior, and new biomarkers are emerging to better stratify these patients. Advanced MOC, instead, is characterized by a poor prognosis. Radical surgery is still the mainstay of treatment since these tumors are chemo-resistant. New targeted therapies are being studied to improve the prognosis in this setting, but unfortunately, none of these have been approved yet. This review focuses on the most relevant clinical, pathological, and molecular features and treatment options according to prognosis.

**Abstract:**

This review includes state-of-the-art prognostic and predictive factors of mucinous ovarian cancer (MOC), a rare tumor. Clinical, pathological, and molecular features and treatment options according to prognosis are comprehensively discussed. Different clinical implications of MOC are described according to the The International Federation of Gynecology and Obstetrics (FIGO) stage: early MOC (stage I-II) and advanced MOC (stage III-IV). Early MOC is characterized by a good prognosis. Surgery is the mainstay of treatment. Fertility-sparing surgery could be performed in patients who wish to become pregnant and that present low recurrence risk of disease. Adjuvant chemotherapy is not recommended, except in patients with high-risk clinical and pathological features. Regarding the histological features, an infiltrative growth pattern is the major prognostic factor of MOC. Furthermore, novel molecular biomarkers are emerging for tailored management of early-stage MOC. In contrast, advanced MOC is characterized by poor survival. Radical surgery is the cornerstone of treatment and adjuvant chemotherapy is recommended, although the efficacy is limited by the intrinsic chemoresistance of these tumors. Several molecular hallmarks of advanced MOC have been described in recent years (e.g., HER2 amplification, distinct methylation profiles, peculiar immunological microenvironment), but target therapy for these rare tumors is not available yet.

## 1. Introduction

Ovarian cancer (OC) is the seventh most-frequent cancer worldwide and the eighth-most-common cause of cancer death in women [1]. The incidence of OC increases with age and is highest in the sixth and seventh decades of life [2], with 239,000 new cases (3.6% of all cancer cases) and 152,000 deaths annually (4.3% of all cancer deaths) [3]. OC includes a highly heterogeneous group of neoplasms, including different histological subtypes with distinct clinical presentations, molecular features, and prognostic outcomes [4]. Epithelial ovarian cancer (EOC) is the most common histological type (90% of all malignant EOC) and, according to Kurman et al. [5], is classified into type 1 tumors, which include low-grade serous carcinoma, endometrioid carcinoma, clear cell carcinoma, and mucinous ovarian carcinoma (MOC), and type 2 tumors, which include high-grade serous carcinoma (HGSOC), carcinosarcoma, and undifferentiated carcinoma. Type 1 EOCs are usually large single cystic neoplasms with an indolent and slow progression. These tumors rarely show *TP53* mutations, have low chromosomal instability, low frequency of homologous recombination defect, and actionable mutations can be present. Conversely, type 2 EOCs show rapid and aggressive progression and poor prognosis. TP53 is frequently mutated and high chromosomal instability and homologous recombination defects are often reported. Compared with type 1 tumors, type 2 EOCs show a high rate of response to chemotherapy but with more recurrences [5]. While HGSOC is the most frequent histological subtype, MOC is sporadic, and its incidence has often been erroneously cited as comprising 5–10% of EOC [6]. However, according to multiple reports, the true proportion of MOC is between 1 and 3% [7]. MOC represents a distinct entity from other histotypes of EOC due to its peculiar behavior, molecular profile, response to chemotherapy, and prognosis compared to the more common HGSOC. 

MOC can be confused with metastatic tumors from other organs, especially gastrointestinal adenocarcinomas. Several clinical algorithms based on the size and laterality of lesions have recently shown good sensitivity to predict the primary origin of MOC [6,8], which is normally characterized by the presence of a large (>10 cm) and unilateral mass in patients aged 20–40 years [9]. Immunohistochemistry and genomic profile may also be useful to confirm the ovarian origin of the mucinous tumor [10]. Normally, the typical immunohistochemical profile of primary MOC is characterized by expression of cytokeratin (CK) 7 and may variably show CK 20, caudal type homeobox 2 (CDX2), estrogen receptor (ER), and progesterone receptor (PgR) positivity. Conversely, paired-box gene 8 PAX8, Wilms’ tumor (WT1), and special AT-rich sequence-binding protein 2 (SATB2) are negative [11]. 

Despite the identification of suggestive clinical parameters and histological characterization of MOCs, the differential diagnosis between primary MOCs and metastatic tumors remains challenging, particularly during the intraoperative assessment of advanced stages [12,13]. A recent study demonstrated that MOC arises from benign and borderline ovarian tumors. Key drivers of progression from benign/borderline lesions to invasive MOC are *TP5* mutation and copy number aberrations, including a notable amplicon on 9p13. Interestingly, a high copy number aberration burden is associated with a worse prognosis in MOC [14].

Primary MOC is generally detected at an early stage, with 83% being diagnosed at stage I and only 17% at stage II or higher [15]. While overall survival (OS) is excellent for early-stage disease [13,16,17], it is drastically worse in the advanced stages [17,18]. These poor survival outcomes are thought to reflect not only inherently aggressive tumor biology but also resistance to currently available chemotherapeutic approaches [17]. 

Several prognostic markers seem to play different roles in early and advanced MOC [18,19]. However, the International Federation of Gynecology and Obstetrics (FIGO) stage system remains the main prognostic factor in MOC patients because the 5-year disease-free survival (DFS) rate is 98% in stage I cases compared to 35% and 14% for stages II through IV [13]. Furthermore, the risk of death when advanced MOC is compared with advanced serous EOC is almost double (hazard ratio—HR: 1.82, 95% confidence interval—CI 1.71–1.94) [19]. 

Given this background, there is an urgent need to identify new novel predictive markers to chemoresponse, and new prognostic factors in MOCs with early recurrence and poor survival. 

This review aims to analyze the different predictive factors of recurrence in these rare tumors, with an emphasis on recent research progress reported in the literature.

## 2. Early MOC

### 2.1. Histopathological Prognostic Factors

In the last two decades, different histopathological features, such as the growth pattern (expansive vs. infiltrative stroma invasion), capsule status (rupture vs. no rupture), and tumor grade, have been described in the literature and correlated with survival outcomes for early-stage MOC [20,21,22,23,24,25]. 

In primary MOC, the need to determine the histological grade and its clinical value has not yet been completely established. To date, two different approaches to assess tumor grade have been recommended. First, the latest World Health Organization classification of gynecological tract tumors does not include any grading schema for this tumor category because the Silverberg system grading, which was based on a combined score, including the predominant architecture (1: glandular, 2: papillary, 3: solid), nuclear atypia (1: mild, 2: moderate, 3: severe) and mitotic activity per 10 high-power fields (1 for 0–9, 2 for 10–24, 3 for ≥25), was recently removed [26]. Second, the latest recommendations from the International Collaboration on Cancer Reporting on ovarian cancer suggest that, if histological grading of MOC is undertaken, the FIGO grading system for endometrioid carcinomas should be used, based on the percentage of the solid component (G1: <5%, G2: between 5 and 50%, G3: >50%), with an upgrade by one in the presence of severe nuclear atypia [27]. Discordant results have been described in the literature concerning the prognostic role of tumor grading in MOC. Ishioka et al. reported that both systems (Silverberg and FIGO systems) have prognostic significance, but that the Silverberg system was superior in predicting the risk of lymph node metastasis [28]. In contrast, two other studies reported that the Silverberg grading and/or FIGO system did not show any prognostic value [23,29]. Recently, Busca et al. compared these two different grading systems and concluded that only Silverberg grading is associated with survival outcomes. Furthermore, they proposed a new binary grading system, named growth-based grading (presence and quantification of infiltrative and expansive growth patterns), that seems to have a good correlation with survival outcomes [30]. However, in primary MOC, the debate on which system should be used to determine the histological grade and its prognostic value remains open. 

In early MOC, the main prognostic histopathological factor seems to be the type of growth pattern (expansive vs. invasive) [31,32,33,34]. In particular, while the MOC infiltrative subtype has an increased rate of local and distant recurrence (peritoneal spread and lymph node involvement) with poor survival outcomes, the MOC expansive subtype has a low metastatic potential and low risk of recurrence. Because the infiltrative pattern is much more frequent in metastatic mucinous non-ovarian cancer, when the tumor shows this characteristic, a secondary neoplasm must be excluded [21,22,23,24,25]. To date, the pattern of tumor invasion, which is one of the most important histological prognostic features described in the literature, is not incorporated into any grading system available. 

Table 1 summarized the main published data on histological features in early stage of primary MOC.

### 2.2. Surgical Prognostic Factors

Surgery (bilateral salpingo-oophorectomy or total abdominal hysterectomy with bilateral salpingo-oophorectomy) represents the cornerstone of treatment for FIGO stage I MOC, and when performed alone, the 5-year survival rate is 90.8%, which is higher than other EOC histotypes [7,35,36,37].

A factor that could be associated with prognosis is the perioperative capsule status. Indeed, capsule rupture in early EOC, which can occur in the pre-operative (spontaneous spill) and intraoperative (surgical spill) settings, upstages patients from IA or IB to IC1 or IC2. Several studies have been published on this topic but its prognostic value in early-stage EOC is still controversial. Vergote et al. showed that a shorter DFS is correlated with capsule rupture, regardless of its occurrence in the pre- or intraoperative setting [38]. In contrast, based on a meta-analysis, including 2382 patients, intraoperative rupture may not decrease progression-free survival (PFS) when compared with no rupture in patients with early-stage EOC [39]. However, Kajiyama et al. underlined that peri- or intraoperative capsule rupture is associated with a poorer oncological outcome in MOC [25]. To date, although the prognostic value of capsule rupture in MOC is not completely proven, surgical spilling should be avoided. 

The assessment of pelvic and/or para-aortic lymph nodes remains the main topic of debate in early-stage MOC. Several studies have reported that surgical lymph node dissection is unnecessary because it does not have a significant impact on OS and DFS [40,41] due to a low presence (from 0% to 2.1%) of lymph node metastasis and, consequently, a low risk of upstaging [42,43,44]. Furthermore, in patients with clinically apparent early MOC who initially received complete surgery without full-staging lymphadenectomy (full peritoneal and clinical lymph node evaluation), a second surgery to evaluate pelvic and para-aortic lymph nodes does not seem to be necessary [41,45]. Gouy et al. confirmed that lymph node assessment could be omitted in the expansive MOC subtype, but this procedure is required for infiltrative MOC cases, during either the initial or restaging surgery [46]. More recently, a study that included 4379 patients from the National Cancer Database with apparent stage IA and IC MOC showed that lymph node metastases were found in 1.2% and 1.6% of patients with stage IA and IC disease, respectively (*p*-value = 0.063). However, they were present in 0.6% of patients with grade 1 tumors, 1.1% of patients with grade 2 tumors, and 5.3% of patients with grade 3 tumors (*p*-value < 0.001). These results confirm that lymph node metastases are rare in these patients but, again, emphasize the prognostic role of tumor grade and suggest considering tumor grading when making decisions regarding the need for lymphadenectomy in early-stage MOCs [47].

The need for appendectomy and its prognostic role in the surgical treatment of early MOC remains another major topic of debate. A recent meta-analysis that included 914 mucinous ovarian neoplasia (borderline and invasive tumors, mostly early stage) estimated histologic appendix tumor involvement in 4.97% of cases, and the pooled odds ratio (OR) showed statistical differences between MOC and borderline tumors (MOC vs. borderline, OR = 2.15; *p*-value ≤ 0.05). The authors suggested an intraoperative evaluation of the appendix, but an appendectomy may be omitted if the appendix appears grossly normal without tumor infiltration [48]. An analysis of 460 MOCs suggests performing appendectomy only in the case of gross abnormalities of the appendix, intraabdominal/peritoneal disease, or ovarian tumor bilaterality [49]. 

In a recent analysis of data from the Surveillance, Epidemiology, and End Results (SEER) cancer registry, 26% of MOCs were diagnosed in women younger than 44 years and a fertility-sparing treatment should be considered in these patients who desire to have future pregnancies [50]. For early MOC, unilateral salpingo-oophorectomy with staging procedures (cytology, peritoneal biopsies, and omentectomy) is a feasible option. The risk of relapse for women with stage I MOC treated with fertility-sparing surgery is low compared with stage I serous cancers (6% vs. 20%, *p*-value ≤ 0.001) [33,51]. Furthermore, the growth patterns in early-stage MOC (infiltrative vs. expansive) do not influence survival outcomes in patients undergoing fertility-sparing surgery [24]. A recent multicenter study on 222 MOCs showed a 5-year PFS rate of 73% (95% CI, 50–86%) for patients who underwent fertility-preserving surgery; however, the authors considered this PFS rate acceptable for women who underwent fertility preservation [52].

### 2.3. Adjuvant Chemotherapy

A large study on 4811 patients diagnosed with stage I MOC identified from the U.S National Cancer Database showed no benefits in the administration of adjuvant chemotherapy (adjusted HR: 1.18, 95% CI: 0.85–1.64) [53]. Similar results were reported in a large study that analyzed FIGO stage IC MOC [54]. Adjuvant chemotherapy does not appear to provide significant benefits, even in patients who have undergone fertility-sparing surgery [55]. Of note, a study of 901 patients affected by stage I MOC who underwent fertility-sparing surgery suggested that adjuvant chemotherapy may increase the risk of death [56]. A study performed on 2041 women with stage I MOC from the National Cancer Database stratified patients into low and high risk based on the following parameters: age, stage, grade, lymphovascular space invasion, and ascites. The risk of death considering patients who had undergone chemotherapy vs. those who had not was similar in low-risk patients (88% vs. 84%; HR =0.80, 95% CI: 0.56–1.15, *p*-value = 0.23) and worse in high-risk patients (51% vs. 74%; HR =1.58, 95% CI: 1.05–2.38, *p*-value = 0.03) [57]. These authors suggested that some patients with high-risk clinical features may benefit from adjuvant chemotherapy [57].

**Figure 1:**
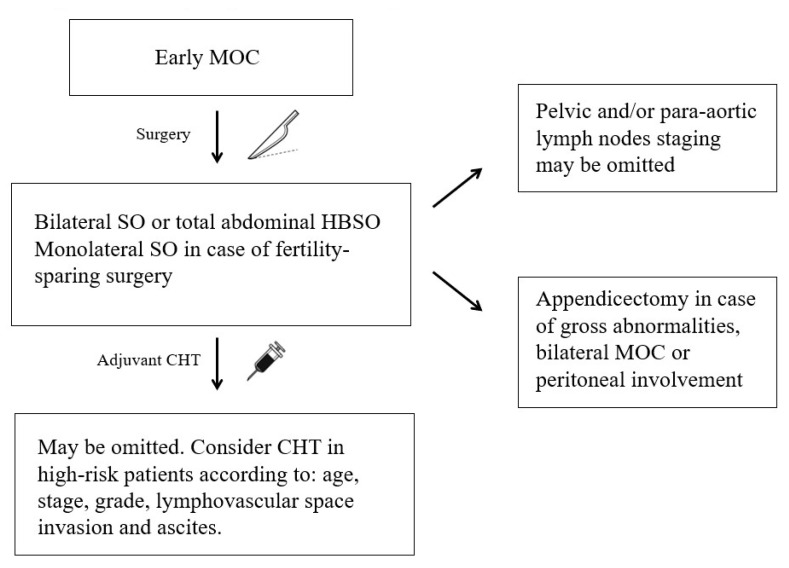
Flowchart of the clinical management of the early-stage MOC according to the most recent evidence (CHT: chemotherapy; HBSO: hysterectomy with bilateral salpingo—oophorectomy; MOC: mucinous ovarian cancer: SO: salpingo—oophorectomy).

A flowchart to guide the management of the early MOC is available in Figure 1.

### 2.4. Molecular Prognostic Factors

Several molecular tumor characteristics in MOC have been recently investigated to find new prognostic biomarkers. In particular, the aberrant expression of different proteins, such as G-protein-coupled receptor 158 (GPR158), CHEK1, FOXM1, KIF23, and PARPBP, are associated with decreased DFS and OS [58,59] but further studies should be performed to translate these molecular features into clinical practice.

Early MOCs are also characterized by specific RNA profiles. A large retrospective study on 257 snap-frozen stage I MOCs biopsies showed high levels of *miR-192/194* [60]. The expression of the *miR-192/194* cluster is directly controlled by wild-type TP53 that, enhancing their transcription, can downregulate genes of G1–G2 phases, targets of these two miRNAs, blocking the cell cycle. In this context, MDM2, a protein that acts as the main inhibitor of TP53, is one of the targets of *miR-192/194.* This positive feedback loop of *TP53-miR-192/194-MDM2* confers a tumor suppressor function to the *miR-192/194* cluster [60,61]. A crucial role in this pathway is also played by *BMI1*, an *miR-194* anticorrelated target gene that acts as a *CDKN2A* protein suppressor [62,63]. *CDKN2A* prevents the degradation and inactivation of p53 operated by MDM2 [60]. Stage I MOCs in this study showed downregulated MDM2 expression and upregulated *CDKN2A* [60]. The authors suggested that *miR-192/194* could be an important target for developing novel therapeutic strategies for MOC [60]. 

Compared to other early EOC histotypes, stage I MOCs showed lower expression of ACVR2B and lower expressions of CDK6 and CDK4, features related to a better prognosis [64]. The role of *miR-192/194* was confirmed by subsequent studies [65,66], which also found new biomarkers for early MOCs: miR-214-3p and let7a-5p are highly expressed, while miR-96 showed low expression levels [65]. High immune reactivity is also noted in stage I MOC compared with other histotypes [65]. Recently, the same group investigated the genome distribution of somatic copy number alterations (SCNAs) in stage I EOC through shallow whole genome sequencing (sWGS), observing three genomic patterns associated with prognosis (stable, unstable, and highly unstable). Most MOC cases were characterized by a lower frequency of regions affected by SCNA (stable or unstable profile) with a good prognosis [67]. 

## 3. Advanced MOC

The advanced stages of MOC have a different landscape compared to the early stage because they have a relative resistance to first-line platinum-based chemotherapy and poor survival outcomes [9,68]. Concerning the differences with serous carcinomas, the 5-year survival rate for patients with stage III serous EOC was 33.6% compared with 25.7% for MOC and, for stage IV disease, was 20.3% and 10.2%, respectively [7]. Furthermore, in a recent meta-analysis including data from seven different studies, the risk of death was almost double in patients with advanced-stage MOC compared to those with serous EOC [19]. 

### 3.1. Histopathological Prognostic Factors

Histopathological prognostic factors in the advanced stage of primary MOC are not completely explored. A recent large retrospective study on 1509 MOCs (1045 stage III and 464 stage IV) showed tumor grading (G1 (21.06 months), G2 (12.22 months), and G3 (11.07 months)), residual disease status (none (23.23 months), <1 cm (12.12months) and >1 cm (6.28 months)), chemotherapy (no (5.06 months) vs. Yes (15.44 months)), stage (III(15.64 months) vs. IV (9.33 months)) and age (<65 years (14.95 months) vs. >65 years (10.61 months)) to be significantly correlated with OS [53]. The worse prognosis of higher-grade MOC was also reported by other authors [30,69].

In another study on 144 metastatic MOCs, the presence of infiltrative invasive pattern, the absence of benign or borderline components, a smaller tumor size, the presence of signet ring cells, and the presence of extracellular mucin were associated with worse survival [70].

Recently, a nomogram to predict cancer-specific survival (CSS) and OS for patients affected by MOC based on clinical, socio-demographic, and histopathological characteristics (age at diagnosis, race, medical insurance, marital status, histological grade, primary side, tumor size, 7th AJCC ovarian cancer stage, TNM stage, and chemotherapy) was developed and could serve as an applicable tool for clinical practice [71].

### 3.2. Surgical Prognostic Factors

The standard surgical treatment of advanced MOC consists of total macroscopic removal of the tumor, i.e., no residual disease (R0). Similarly to serous advanced OC, several authors reported that optimal debulking is also related to better survival for MOC [17,72,73]. Debulking surgery with the objective of a macroscopically complete resection remains the goal of surgical treatment.

Over the last decade, one of the most intriguing methods adopted for the treatment of advanced EOC is represented by hyperthermic intraperitoneal chemotherapy (HIPEC). In a recent metanalysis, patients treated with HIPEC at the time of cytoreductive surgery showed better OS and PFS compared to patients who did not undergo HIPEC (HR = 0.54, 95% CI: 0.45 to 0.66, HR = 0.45, 95% CI: 0.32 to 0.62, respectively) [74]. However, the role of HIPEC for MOC treatment is unclear, as this histotype is underrepresented in trials [75,76]. To date, a retrospective analysis including 77 patients with MOC and peritoneal carcinomatosis treated with debulking surgery and HIPEC showed an interesting 5-year OS and DFS of 69.6% and 53.8%, respectively [77], but prospective data on the role of HIPEC for these rare tumors are needed.

### 3.3. Adjuvant Chemotherapy

Advanced MOC is a chemoresistant tumor; however, in a large study including stage III-IV MOCs, patients who received chemotherapy showed better OS compared to those who did not (median OS 15.44 vs. 5.06 months, *p*-value ≤ 0.001) [53].

Due to the biological and molecular similarities of MOC and mucinous colorectal cancer, chemotherapy protocols for colorectal cancer have been proposed as an alternative to the standard carboplatin–paclitaxel regimens usually used for EOC. Unfortunately, the Gynecology Oncology Group trial 241, which was designed to explore the benefit of a colorectal chemotherapy regimen in newly diagnosed MOC, was prematurely stopped for slow and poor accrual. Data from the 50 recruited patients showed no statistically significant differences in PFS and toxicity profiles between the treatment arms [78]. 

**Figure 2:**
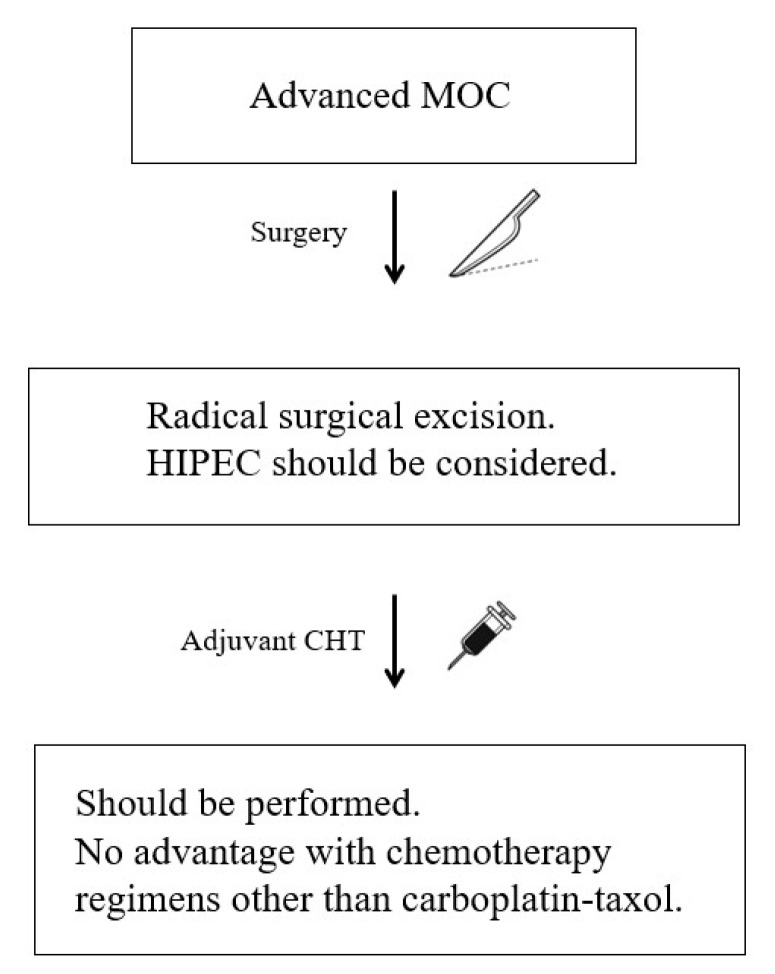
Flowchart of the clinical management of advanced MOC according to the most recent evidence (CHT: chemotherapy; HIPEC: hyperthermic intraperitoneal chemotherapy; MOC: mucinous ovarian cancer).

A flowchart to guide the management of advanced MOC is available in Figure 2.

### 3.4. Molecular Prognostic Factors

Chemoresistance of MOC to the standard treatments prompts interest in the development of targeted molecular therapies. Unfortunately, *C-myc* overexpression [79], *KRAS* mutations [80], *HER-2* gene amplification [81], and *TP53* mutations [82] do not seem to be correlated with tumor prognosis, and no alternative therapeutic options to the conventional chemotherapy regimen are available at the moment. A recent study analyzed clinical, pathologic, and gene-expression data in tumor samples from a large cohort of patients with MOC; the authors found that increased expression of *THBS2* and *TAGLN* is associated with shorter OS (HR: 1.25; 95% CI, 1.04–1.51, *p*-value = 0.016 and HR: 1.21; 95% CI, 1.01–1.45, *p*-value = 0.043), respectively, and is related to infiltrative subtypes. These authors observed an elevated expression of HER2 in the early stage and expansive pattern MOC [83].

Another study evaluated the prognostic role of ciliated cell markers expressed in 118 high-grade EOCs by comparing them with normal tubal and endometrial tissue, cystadenomas, and borderline and low-grade ovarian tumors. The expression of ciliated cell markers was associated with a better prognosis and decreased with increasing grade, presence of metastasis, and mucinous histotype [84].

Recently, the role of polo-like kinase 1 (PLK1) in MOC biology has been investigated. In a study of MOC cell lines, PLK1 downregulation resulted in interference in cancer cell growth [85]. When translated into a mice xenograft model, the combination of onvansertib (PLK1 inhibitor) and paclitaxel resulted in a mitotic block and apoptosis induction with prolonged survival [84]. 

Phosphatidylinositol 3-kinase PI3K pathway aberrations were described in serous EOC [86,87]. PI3K alterations occur with *KRAS* mutations, and in MOC cell lines with *KRAS* mutation PI3K and MEK (mitogen-activated protein kinase), inhibitors show synergistic activity [88]. Given these premises, encouraging results were reported in a phase I study that combined MEK and PI3K inhibition in patients with KRAS-mutated EOC [89].

MOC is also characterized by distinct methylation profiles. MOC showed that a dominant network module is the proteasome subunit beta (PSMB) family. In particular, combined functional module and methylation analysis identified PSMB8 as a candidate marker for MOC, suggesting a potential role of proteasome inhibitors such as carfilzomib [90].

Regarding the immunology of EOC, evidence indicates that these tumors express a multitude of known tumor-associated and mutational antigens. Furthermore, tumor-infiltrating lymphocytes (TILs) have been described in several EOCs, correlating with improved survival outcomes [91,92,93]. However, despite a preclinical rationale, immunotherapy has yielded modest results in the treatment of EOC [94,95]. In this context, the immunological microenvironment of MOC has been poorly investigated; however, a recent study described the immune landscape for a large series of MOC. Most of the immune infiltrate is located in the stroma of the tumor compared to the epithelium, and advanced MOCs exhibit greater epithelial infiltration by programmed death-ligand 1 (PD-L1)-negative macrophages and lower PD-L1-positive macrophages compared with stage I/II cancers (*p*-value = 0.004 and *p*-value = 0.014, respectively). The high epithelial density of FOXP3+ cells, CD8+/FOXP3+ cells, and PD-L1- macrophages is related to poor survival. The authors also concluded that almost all MOCs (about 90%) are immunogenically ‘cold’, suggesting, also for MOC, a limited role for immunotherapy [96]. Nevertheless, 15–20% of MOCs have defects in the mismatch-repair DNA pathways, leading to microsatellite instability and high mutation burdens [97,98,99]. MOCs with these features may benefit from immunotherapy.

## 4. Conclusions

MOC is a rare entity frequently diagnosed at an early stage. The pattern of growth (expansive vs. invasive) is the main prognostic factor but it is not incorporated in any classification system. Early-stage MOC can be treated with hysterectomy and bilateral salpingo-oophorectomy or bilateral salpingo-oophorectomy alone. Nodal involvement is rare; thus pelvic and/or para-aortic lymphadenectomy is not recommended. Appendectomy should be performed only in the case of gross involvement, bilateral MOC, or peritoneal involvement. Adjuvant chemotherapy does not appear to affect prognosis, although a recent study suggests its use in selected cases. The advanced stage of MOC requires adjuvant treatment but it is characterized by an intrinsic chemoresistance with a poor prognosis. To date, no better treatment options are available than the standard platinum-based chemotherapy. The ongoing improvement in molecular profiling of MOC will hopefully allow for the discovery of novel and targetable oncogenic drivers. 

Further studies should be performed to comprehensively characterize this rare disease and improve its treatment and outcomes.

## Figures and Tables

**Table 1:** Summary of the main published data on histological features in early stage of primary MOC.

Study	Growth Pattern	Number of Early MOC Patients	FIGO Staging, Number	Tumor Grading, Number	Rupture Capsule	Recurrence Rate	FU (Months)
Lee and Scully 2000 [20]	ESI ISI	12 6	Ia Ia	G1: 8, G2: 4 NA	IO: 3/25 *	0/12 1/6(16.7%)	33–135 36–135
Chen et al. [21]	ISI	4/6	Ia, 2 Ic, 2	G1:1 G2: 3	PO:1 IO: 1	1/2 1/2	9–161
Tabrizi et al. [22]	ESI ISI	26 4	NA NA	NA G3: 4	PO: 5 IO: 12	6/26 1/4	6–176
Muyldermans et al. [23]	ESI ISI	21 12	Ia, 11 Ic, 10 Ia, 9 Ic, 3	G1: 20 G2: 20 G3: 4	NA NA	0 1/9 1/3	64
Gouy S et al. [24]	ESI ISI	29 39	Ia, 13 Ic1, 9 Ic2, 5 Ic3, 2 Ia, 22 Ic1, 9 Ic2, 7 Ic3, 1	G1: 11 G2: 10 G3: 0 Gx: 8 NA	PO:6 IO: 8 PO:6 IO: 6	3/29 6/39	NA
Kajiyama et al. [25]	NA	194	Ia, 85 Ib, 2 Ic1, 58 Ic2, 18 Ic3, 31	NA	IO: 58 PO: 49	36/194	2–248
Rodriguez and Prat [31]	ESI ISI	15 11	Ia, 10 Ic, 5 Ia, 8 Ic, 3	G1:4, G2:11 G3: 11	PO: 4/15 IO: 1/15 PO: 1/11 IO: 2/11	0/15 0/8 3/3(100)	40–194 12–180
Ludwick et al. [34]	ESI	3	Ia:3	NA	NA	3/3	7–32

DOT: dead of tumor, FU: follow-up, ESI: expansile stromal invasion, Gx: undetermined grade IO: intraoperative, ISI: invasive stromal invasion, NED: no evidence of disease, NA: not available, OS: overall survival, PO: perioperative. * Subtype of infiltration in these patients is unknown.
